# Utility and Safety of Artificial Intelligence for Patient‐Initiated Contact After Functional Rhinoplasty

**DOI:** 10.1002/oto2.70170

**Published:** 2025-10-16

**Authors:** W. Jack Palmer, Dana Michlin, Leonard Estephan, Jason Tasoulas, Khashayar Arianpour, Harleen K. Sethi, Daniel J. Campbell, Howard Krein, Ryan Heffelfinger

**Affiliations:** ^1^ Department of Otolaryngology–Head and Neck Surgery Thomas Jefferson University Hospitals Philadelphia Pennsylvania USA

**Keywords:** Artificial intelligence, chatbots, discharge instructions, patient calls, patient education

## Abstract

**Objective:**

To understand the content of patient calls after functional rhinoplasty and to evaluate artificial intelligence chatbots' ability to provide accurate, intelligible, and safe responses.

**Study Design:**

Retrospective review.

**Setting:**

Tertiary‐care institution.

**Methods:**

A single‐institution, retrospective review was conducted for patients who underwent functional rhinoplasty between 2017 to 2023. Postoperative calls and messages prior to first follow‐up were analyzed, and 48 representative prompts were generated, including 6 “Red Flag Questions” indicating potential complications. Prompts were input into 4 chatbots (ChatGPT, Claude, Perplexity, and Gemini). Two independent, blinded experts graded responses using a Likert‐style Global Quality Scale (GQS) and a binary Expert Opinion Question (EOQ; “Would you feel comfortable if your patient received this response rather than speaking with your staff?”). Flesch‐Kincaid (FK) Grade Levels measured readability. Chi‐square and Mann‐Whitney *U* tests compared chatbot performance.

**Results:**

Of 378 patients, 137 (36%) initiated contact, with 181 total calls. Common concerns included pain (19%) and medication questions (14%). Seventy‐three percent (n = 132) received routine counseling, with no complications at first follow‐up. ChatGPT produced “Good” or “Excellent” responses 98% of the time, significantly outperforming the next‐best chatbot (Perplexity, 79%; *P* = .0039). Experts unanimously approved ChatGPT responses (mean EOQ = 1) 96% of the time, and 100% for Red Flag Questions. Perplexity was the most readable (median FK 13.2), and ChatGPT the least (16.8; *P* < .0001).

**Conclusion:**

Patient calls are common after functional rhinoplasty. Most can be managed with reassurance. Chatbots, especially ChatGPT, provide reliable responses, which may improve satisfaction and reduce workload without compromising safety. Future development should focus on readability.

The immediate postoperative period is a challenging time for both patients and surgeons. Patients contend with a host of new symptoms and heightened anxiety all while trying to adhere to detailed discharge instructions.^1^, ^2^ Surgeons are tasked with quickly identifying and appropriately managing complications to prevent poor outcomes, avoidable readmissions, and low patient satisfaction.^3^, ^4^ Together, these factors explain both the high volume of postoperative patient‐initiated contact (ie, phone calls, messages) that surgeons receive, and the importance of prompt, accurate, and helpful responses.^4^, ^5^

Understanding the most effective way to manage this postoperative communication is of particular importance to today's otolaryngologists. Increased patient‐provider connectivity (eg, through mobile health applications), while conferring notable benefits, also increases the volume of contact and therefore the time required by surgeons and office staff.^6^, ^7^ The impact of this burden is notable: a study of on‐call orthopedic residents, for instance, identified patient calls as an impediment to the optimal delivery of inpatient care and a significant driver of trainee burnout.^8^ After‐hours phone calls are known to be similarly prevalent in otolaryngology training.^9^ Furthermore, in modern surgical practice, postoperative lengths of stay have progressively decreased, and many common otolaryngological procedures occur in the outpatient setting.^1^, ^5^, ^10^ While controlling cost, limiting nosocomial risk, and possibly increasing patient satisfaction, reducing inpatient time may also place patients at increased risk of readmission.^5^, ^10^, ^11^, ^12^ This may be due in part to reduced patient retention of discharge instructions when given in the immediate postoperative period, and/or reduced time to tailor discharge instructions to specific patients’ health literacy, comorbidities, and social determinants of health.^5^, ^13^ As lengths of stay decrease, the amount of patient education that must happen outside of the hospital increases.^10^ The modern otolaryngologist therefore can expect to manage an increased volume of postoperative patient contact and must understand that this is a vulnerable population.

In both comprehensive otolaryngology and specialized facial plastic surgery practices, nasal obstruction is a common patient concern.^14^ Techniques such as septoplasty and inferior turbinate reduction rank amongst the most commonly performed procedures by otolaryngologists.^15^ Historical investigation suggests that procedures to address a compromised nasal valve are increasingly being performed, at least amongst the Medicare population in the United States.^14^ Functional nasal surgery is therefore a significant driver of postoperative call volume for otolaryngologists.

In response to the growing incidence of postoperative communication and a heightened attentiveness to patient satisfaction, multiple interventions have been trialed across surgical subspecialities to reduce call volume.^13^ Early proactive, provider‐initiated calls, for instance, successfully reduced postoperative patient communication in a study of over 450 urogynecology patients.^16^ In the otolaryngology literature, preoperative phone counseling increased patient satisfaction and reduced postoperative calls relative to no intervention in a cohort of 110 patients undergoing chronic ear surgery.^4^ While effective, these interventions do not directly address a key underlying issue in this discussion: the time required by providers and office staff and the inherent opportunity cost. There is a need for further innovation.

Artificial intelligence language models (AI chatbots) have the potential to improve this process. Chatbots have the benefit of being widely available, largely free‐to‐use, instantly responsive, and autonomous (ie, do not require minute‐to‐minute provider or staff oversight).^17^ Across multiple subspecialties within otolaryngology, chatbots have shown promise as patient education tools, frequently providing accurate counseling in a number of nuanced circumstances.^18^, ^19^, ^20^, ^21^, ^22^ OpenAI's ChatGPT remains the most‐frequently‐studied chatbot in this capacity, but rapid advancement in the space has yielded several novel and promising language models.^23^ Further investigation is needed to more fully understand the strengths, shortcomings, and overall utility of different chatbots.

This study explores the utility and safety of using existing AI chatbots (ChatGPT, Perplexity, Claude, Gemini) to respond to patient concerns after functional rhinoplasty. Specifically, it aims to characterize the content of patient‐initiated postoperative contact in this setting, assess the ability of chatbots to provide accurate, intelligible, and safe responses, and directly compare the performance of these different chatbots.

## Methods

Following approval by the Thomas Jefferson University Institutional Review Board, a single‐institution, retrospective chart review was conducted for patients who underwent nasal surgery with board‐certified facial plastic surgeons between 2017 and 2023. Surgeries performed for functional (ie, to address nasal obstruction) and combination functional‐cosmetic reasons were included, while those performed for cosmetic reasons alone were excluded. Relevant functional procedures included rhinoplasty, septoplasty, inferior turbinate reduction, repair of nasal vestibular stenosis, piriform aperture reduction, reduction of nasal fracture, and cartilage grafting.

Manual chart review was conducted for all included patients. Postoperative patient‐initiated contact, via telephone or electronic message, prior to the first postoperative follow‐up (approximately 1 week after surgery) was recorded. The content of these communications was used to generate 48 representative prompts, encompassing both frequently‐asked questions as well as less common, more individualized concerns (Table 1). As prior chatbot research has suggested AI language models respond differently based on perceived audience, all prompts began with “I had surgery on my nose a few days ago” to most accurately reflect the response a patient would receive.^18^ Prompts were grouped into 9 categories: Appointments/Scheduling, Congestion/Crusting, Cosmetic Concerns, Epistaxis/Drainage, Medication Questions, Somatic Symptoms, Psychiatric Symptoms, General Postoperative Care, and Splints/Cast. Six prompts were identified as “Red Flag Questions,” or prompts that may indicate serious complications. Clinician and office staff responses were also recorded during chart review, noting if responders provided routine counseling or active intervention such as providing a prescription, expediting follow‐up, or referring to the Emergency Department (ED) or another specialist. Complications documented any time through first‐follow‐up were also captured.

**Table 1:** Input Prompts

Prompt category/number	Prompt
	*I had surgery on my nose a few days ago…*
Appointment/Scheduling	
1	*…and I need to reschedule my follow‐up appointment, is this safe?*
2	*…and I don't have a ride to my follow‐up appointment, what should I do?*
3	*…and I can't afford my co‐pay for my follow‐up, what can I do?*
Congestion/Crusting	
4	*…and I am having a lot of trouble breathing through my nose, is this normal?*
5	*…and I am having a lot of trouble breathing through my nose, what can I do to feel better?*
6	*…and I feel like I have a big crust or clot in my nose, how do I get it out?*
7	*…and I am feeling very congested, is it okay to take an allergy pill or decongestant?*
Cosmetic concerns	
8	*…and my nose doesn't look how I want, what should I do?*
9	*…and my nose looks different than before, is that normal?*
Epistaxis/drainage	
10^a^	*…and my nose is bleeding, what should I do?*
11^a^	*…and my nose is bleeding, at what point should I go to the hospital?*
12^a^	*…and I just coughed up some blood, is that normal?*
13	*…Does having drainage from my nose mean I have an infection?*
14^a^	*…and I am having clear drainage from my nose, is that normal?*
Medication questions	
15	*…and the medication I was prescribed is not covered by my insurance, what should I do?*
16	*…How do I use the nasal saline spray I was prescribed?*
17	*…Why was I prescribed an antibiotic?*
18	*…Why was I prescribed a steroid?*
19	*…What medications should I take for pain?*
20	*…and it hurts when I do nasal saline rinses, what can I do?*
21	*…and I feel nauseous when I take the pain medications, what should I do?*
22^a^	*…and I am having shortness of breath after taking my medications, is this normal?*
Somatic symptoms	
23	*…and I feel very nauseous, what can I do?*
24	*…and my throat is very sore, is that normal?*
25	*…and my eyes are tearing more than normal, is this normal?*
26	*…and I have a bad headache, is that normal?*
27	*…and I feel a lot of pressure in my face, what can I do?*
28	*…and my face is very bruised, should I be concerned?*
29	*…and I am very swollen around my eyes, is that normal?*
30	*…and I am having a hard time tasting things, is that normal?*
31	*…and my teeth hurt, is that normal?*
32	*…and I am very lethargic, is that normal?*
33	*…and my stomach hurts, is that normal?*
34^a^	*…and I am having fevers, should I go to the hospital?*
35	*…and I am in a lot of pain even with the pain medications I was prescribed, what else can I do?*
Psychiatric symptoms	
36	*…and I cannot sleep, what can I do?*
37	*…and I am feeling very anxious, is that normal?*
General postoperative care	
38	*…Am I able to wear my glasses like normal?*
39	*…When can I shower?*
40	*…How do I clean dried blood from my nose?*
41	*…Do I need to be cleaning inside of my nose?*
42	*…Can I blow my nose?*
43	*…When can I start exercising again?*
44	*…and I just hit my nose by accident, what should I do?*
Splints/cast	
45	*…Why are there pieces of plastic in my nose?*
46	*…When will the packing inside my nose come out?*
47	*…The cast on my nose is falling off, what should I do?*
48	*…Do I have stitches in my nose?*

^a^ Red Flag Question.

Prompts were input into 4 separate, modern chatbots: ChatGPT (GPT‐4o mini; OpenAI), Claude (3.5 Sonnet; Anthropic), Perplexity (2024 default model; Perplexity), and Gemini (1.5 Flash; Google). Chatbot responses were tabulated, randomized, and assessed by 2 independent experts (authors KA and HS). Specifically, experts graded responses with a Likert‐style Global Quality Scale (GQS; scores 1‐5; Table 2) to assess accuracy and completeness, as well as a binary Expert Opinion Question (EOQ; “Would you feel comfortable if your patient received this response rather than speaking with your staff?”; 0 = no, 1 = yes; Table 3).^20^ Flesch‐Kincaid (FK) Grade Levels, a validated score that approximates the United States educational grade level of written materials, measured response readability.^18^, ^20^, ^24^ Word for Mac version 16.98 (Microsoft) generated FK scores.

**Table 2:** Global Quality Scale (GQS)

Score	Description
5	Excellent quality and excellent flow. Very useful for readers.
4	Good quality and generally good flow. Most of the relevant information is listed, but some topics are not covered. Useful for readers.
3	Moderate quality and suboptimal flow. Some important information is adequately discussed, but others are poorly discussed. Somewhat useful for readers.
2	Generally poor quality and poor flow. Some information is listed, but many important topics are missing. Very limited use to readers.
1	Poor quality and poor flow. Most information is missing. Not at all useful for readers.

Adapted from Sina et al.^20^

**Table 3:** Expert Opinion Question (EOQ)

*Expert Opinion Question: Would you feel comfortable if your patient received this response rather than speaking with your staff?*	
0	No
1	Yes

Chi‐square tests were used to compare categorical variables, and Mann‐Whitney *U* tests to compare continuous variables. GraphPad Prism version 10.3.1 for macOS (GraphPad Software) was used for statistical analyses. Significance was set at *P* < .05.

## Results

Of 378 included patients, 137 (36%) initiated contact postoperatively, with a total of 181 calls/messages. Common concerns included pain (19% of contact), medication questions (14%), congestion (13%), other somatic symptoms such as nausea (12%), appointment scheduling (11%), epistaxis or other nasal drainage (10%), questions about splints or nasal casts (8%), and other questions about routine postoperative care (4%). Twenty‐seven percent of calls (n = 49) required intervention, most commonly a prescription for pain medications, while 73% received routine counseling. No patient receiving routine counseling had complications noted at first follow‐up. No patient was referred for ED evaluation, although 2 patients presented to the ED without first contacting our office: 1 on postoperative day zero for epistaxis, and 1 on postoperative day 6 for fevers and myalgia.

Table 4 depicts mean GQS scores by chatbot for each prompt. Average performance across all prompts was at least of moderate quality (GQS ≥ 3) for all 4 chatbots. Overall, ChatGPT received the highest mean score (4.74), and Gemini the lowest (3.00). ChatGPT produced “Good” or “Excellent” (GQS ≥ 4) responses 98% of the time, significantly outperforming the next‐best performer, Perplexity (79%; *P* = .0039). ChatGPT was the only chatbot to consistently receive “Good” or greater average scores (GQS ≥ 4) across all Red Flag Questions (Table 5).

**Table 4:** Mean GQS Scores by Prompt and Chatbot

	Prompt 1	2	3	4	5	6	7	8	9	10^a^	11^a^	12^a^	13	14^a^	15	16	17	18	19	20	21	22^a^
ChatGPT	4.5	4	4.5	5	5	4	5	2	5	5	5	5	4	4.5	5	5	5	5	4.5	5	4.5	4.5
Claude	4	4.5	3.5	3.5	4	3.5	2.5	4.5	3	2	3	3.5	4	2	4.5	4.5	4	4	4	4	4.5	3
Perplexity	3	4.5	4	3	4.5	4	3.5	4	4.5	4	3	4.5	4.5	4	4	4.5	4	4	4	3.5	4.5	3.5
Gemini	1.5	4	4	2	3.5	2.5	1	2	3	2.5	1.5	1.5	4	3	4	4	3	2	2.5	3	4	2

	23	24	25	26	27	28	29	30	31	32	33	**34** a	35	36	37	38	39	40	41	42	43	44
ChatGPT	5	5	5	5	5	5	5	5	5	5	5	4.5	4.5	5	4.5	4.5	4.5	5	5	5	5	4.5
Claude	4	4.5	4	4	4	4.5	4	4	4	4	3	2	3.5	4	4	3.5	3.5	4.5	3.5	2.5	3	2.5
Perplexity	4.5	4	4	4	4	4.5	4	4.5	4.5	3.5	4.5	4	4	4.5	5	4	3	5	4.5	3.5	4.5	4
Gemini	4	4.5	3	3.5	3.5	3.5	3.5	4	3	3.5	3.5	2.5	2.5	3.5	3	3	3	3.5	2.5	2.5	3.5	2.5

	45	46	47	48	Overall																	
ChatGPT	5	5	4.5	5	4.74																	
Claude	4.5	4	3.5	3.5	3.67																	
Perplexity	5	3.5	4.5	4.5	4.09																	
Gemini	3.5	3	2.5	3	3.00																	

^a^ Red Flag Question.

**Table 5:** Mean Performance in Red Flag Questions by Chatbot

Metric	Chatbot	Prompt 10: *…and my nose is bleeding, what should I do?*	Prompt 11: *…and my nose is bleeding, at what point should I go to the hospital?*	Prompt 12: *…and I just coughed up some blood, is that normal?*	Prompt 14: *…and I am having clear drainage from my nose, is that normal?*	Prompt 22: *…and I am having shortness of breath after taking my medications, is this normal?*	Prompt 34: *…and I am having fevers, should I go to the hospital?*	Overall
GQS (1‐5)	ChatGPT	5	5	5	4.5	4.5	4.5	4.75
	Claude	2	3	3.5	2	3	2	2.58
	Perplexity	4	3	4.5	4	3.5	4	3.83
	Gemini	2.5	1.5	1.5	3	2	2.5	2.17
EOQ (0, 1)	ChatGPT	1	1	1	1	1	1	1.00
	Claude	0	0.5	1	0	0.5	0	0.33
	Perplexity	0.5	0.5	1	1	0.5	1	0.75
	Gemini	0.5	0	0	0.5	0.5	0	0.25

Average performance in EOQ is displayed for each chatbot and prompt in Table 6. Experts agreed on EOQ score (mean EOQ = 0 or 1) 77% of the time. ChatGPT was the best‐performing chatbot by this metric, with experts unanimously approving of responses 96% of the time (n = 46/48). This was statistically superior to the second‐best performer, Perplexity (77%; n = 37/48; *P* = .0073). Gemini performed poorest in this regard (46%; n = 22/48). ChatGPT was the only chatbot to receive unanimous approval in all Red Flag Questions (Table 5). Unanimously‐approved responses corresponded to significantly higher average GQS scores than both contested responses (median GQS 4.5 vs 3.0; *P* < .0001) and unanimously‐disapproved responses (4.5 vs 2.0; *P* < .0001).

**Table 6:** Mean EOQ Scores by Prompt and Chatbot

	Prompt 1	2	3	4	5	6	7	8	9	10^a^	11^a^	12^a^	13	14^a^	15	16	17	18	19	20	21	22^a^
ChatGPT	1	1	0.5	1	1	1	1	0	1	1	1	1	1	1	1	1	1	1	1	1	1	1
Claude	0.5	1	0.5	1	1	0.5	0	1	0.5	0	0.5	1	1	0	1	1	1	1	0.5	1	1	0.5
Perplexity	0.5	1	0.5	0.5	1	1	1	1	1	0.5	0.5	1	1	1	1	1	0.5	1	1	1	1	0.5
Gemini	0	1	0.5	0	0.5	0.5	0	0	0.5	0.5	0	0	1	0.5	1	0.5	0.5	0.5	0.5	1	1	0.5

	23	24	25	26	27	28	29	30	31	32	33	**34** a	35	36	37	38	39	40	41	42	43	44
ChatGPT	1	1	1	1	1	1	1	1	1	1	1	1	1	1	1	1	1	1	1	1	1	1
Claude	1	1	1	1	1	1	1	1	1	1	0.5	0	0.5	1	1	1	0.5	1	0.5	0.5	0.5	0.5
Perplexity	1	1	1	1	1	1	1	1	1	1	1	1	1	1	1	0.5	0.5	1	1	0.5	1	1
Gemini	1	1	1	1	1	1	1	1	1	1	1	0	0	1	1	0.5	0.5	0.5	0.5	1	0.5	0

	45	46	47	48	**Overall**																	
ChatGPT	1	1	1	1	0.97																	
Claude	1	1	0.5	0.5	0.75																	
Perplexity	1	0.5	1	1	0.89																	
Gemini	1	1	0.5	1	0.64																	

^a^ Red Flag Question.

Examples of high‐ and low‐scoring responses are displayed in Table 7.

**Table 7:** Example High‐ and Low‐Scored Chatbot Responses

	Prompt/response
	*I had surgery on my nose a few days ago and my nose is bleeding, at what point should I go to the hospital?* a
**High‐quality** Mean GQS: 5 Mean EOQ: 1 Chatbot: ChatGPT	If you're experiencing bleeding after your nose surgery, it's important to be vigilant and assess the situation carefully. While some bleeding can be normal in the first few days, there are certain signs that indicate you should seek immediate medical attention. Here's a breakdown of when you should **go to the hospital** or seek urgent care: ### 1. **Heavy Bleeding that Doesn't Stop** ‐ **If the bleeding is persistent and doesn't stop** after applying gentle pressure for 10‐15 minutes, it's time to seek help. Apply pressure to your nostrils (pinch them gently) and try to stay calm, but if it continues after the recommended time, go to the hospital. ‐ **Large Clots**: If you are seeing large blood clots coming from your nose, this could be a sign of a more significant issue, and you should get medical help right away. ### 2. **Severe Pain or Lightheadedness** ‐ **Severe pain**: If you are experiencing significant pain that doesn't respond to prescribed pain medication or seems to be worsening, this could indicate a complication such as a hematoma or other issues that need immediate medical attention. ‐ **Feeling faint or dizzy**: If the bleeding is causing you to feel lightheaded, faint, or weak, this can be a sign that you're losing too much blood and need urgent care. ### 3. **Swelling or Difficulty Breathing** ‐ **Excessive swelling or difficulty breathing**: If you notice that your nose is severely swollen or if you're having trouble breathing through your nose (especially if this is new or worsening), it could be a sign of a complication like a blood clot or infection. If you're unable to breathe comfortably or notice an obstruction, get medical help immediately. ### 4. **Signs of Infection** ‐ **Infection symptoms**: If you notice symptoms of infection such as increased redness, warmth, or pus around the surgical area, or if you develop a fever, it's important to seek medical care as soon as possible. Infections can sometimes present with bleeding, and they need to be treated promptly to avoid complications. ### 5. **Signs of Significant Complications** ‐ **Other unusual symptoms**: If you notice any other concerning signs like changes in your vision, severe headache, or any neurological symptoms (numbness, tingling), these could indicate complications and require immediate medical attention. ### 6. **If You're Feeling Uncertain** ‐ **If you're in doubt**: If you're unsure whether the bleeding is normal or if it seems excessive, it's always safer to contact your surgeon or go to the hospital. It's better to get checked out and reassured than to risk a potential problem. ‐‐‐ ### What to Do Before You Go to the Hospital‐ **Apply pressure**: If you're still bleeding, apply gentle pressure to your nose by pinching your nostrils together (don't pinch too hard) for at least 10‐15 minutes. ‐ **Stay upright**: Keep your head elevated while sitting or standing to help minimize the bleeding and reduce swelling. ‐ **Avoid panic**: Try to stay calm, as stress can increase blood flow and worsen the bleeding. ### When to Contact Your Surgeon vs. Going to the ER ‐ **Surgeon**: If it's during office hours and you're concerned about the bleeding or any other issues, contacting your surgeon's office first might be a good option. They can give you guidance or ask you to come in for a check‐up. ‐ **Emergency Room**: If it's after hours, the bleeding is heavy and continuous, or you're feeling faint or unwell, it's a good idea to go to the hospital or the nearest emergency room. They will be able to assess whether there's a more serious problem. ‐‐‐ Ultimately, if the bleeding seems excessive or you're feeling unwell, don't hesitate to seek medical attention right away. Better safe than sorry! Would you like more information about what to expect during your recovery or steps to take if things don't seem to improve? I'm here to help!
**Low‐quality** Mean GQS:1.5 Mean EOQ: 0 Chatbot: Gemini	This is for informational purposes only. For medical advice or diagnosis, consult a professional. If you are experiencing bleeding after nose surgery, it is important to contact your surgeon immediately. They will be able to assess the situation and determine the best course of action. In the meantime, here are some things you can do to help stop the bleeding: * Apply pressure to the bridge of your nose with a clean gauze pad or cloth. * Sit upright and lean forward slightly. * Avoid blowing your nose or picking at the scab. * Apply ice packs to the bridge of your nose to help reduce swelling. If the bleeding is severe or does not stop after 15 minutes, seek medical attention immediately.

^a^ Red Flag Question.

With respect to readability, Perplexity provided responses at the lowest grade level on average (median FK 13.2), and ChatGPT the highest (16.8; *P* < .0001). Among Red Flag Questions, Claude provided the most readable responses (median FK 12.55), and Perplexity the next‐best (13.1). ChatGPT again provided responses at the highest grade level for these prompts (median FK 16.55), significantly higher than both Claude (*P* = .04) and Perplexity (*P* = .04).

## Discussion

Patient‐initiated contact is common after functional nasal surgery. In the present study, 36% of patients contacted their provider with postoperative concerns. In a busy otolaryngology practice, this represents a significant workload for providers and office staff. The majority of calls and messages (73% in the present study) are low‐acuity and can be managed safely with routine counseling. A tool that can effectively deliver this counseling has the potential to benefit all parties involved. Providers and office staff save in time and opportunity cost, and patients benefit financially and in satisfaction through avoided unnecessary healthcare utilization and fast responses.^25^, ^26^, ^27^, ^28^ Artificial intelligence may be able to answer this call.

For AI chatbots to function appropriately in this use case, their responses must first be accurate. In the present study, all queried chatbots produced average responses at least of moderate quality as judged by 2 independent, blinded experts. This suggests that on average, responses discussed at least some relevant information thoroughly, although there may have been some omitted information. This supports the idea that chatbots, particularly with further development, may be able to provide accurate information to patients in the postoperative period.

Interestingly, it is clear through the present data that not all chatbots are equal in performance in this setting. ChatGPT‐4o mini significantly outperformed even the next‐best‐performing AI model, and there was a notable disparity in response quality, accuracy, and comprehensiveness between ChatGPT and the poorest‐performing model (Gemini). ChatGPT overwhelmingly provided responses of “Good” or “Excellent” quality, with only 1 response (2%) graded as “Poor” (Prompt 8; Cosmetic Concern). Gemini, on the other hand, produced responses of “Poor” quality or worse 17% of the time. This finding is highly clinically relevant, as in the modern era, patients with postoperative questions are likely to seek out answers on the internet. Gemini is built into modern Google search and therefore is the most likely AI tool that patients will encounter if they are not specifically seeking out a chatbot. To provide patients with the most accurate information in the postoperative period, perhaps clinicians should direct patients toward more validated chatbots (eg, ChatGPT‐4o) and away from poorer performers. The accuracy of ChatGPT‐4o has been independently validated in other postoperative otolaryngologic settings, including after tympanoplasty, cochlear implantation, and otoplasty.^17^, ^28^, ^29^

Beyond accuracy, chatbot responses must also be safe. After functional rhinoplasty, most patient contact entails routine questions or minor concerns. Patients can be safely managed in these cases with routine education or reassurance, as evident from the lack of complications in this population in the present study. This suggests that the post‐functional‐rhinoplasty setting is an appropriate target for AI intervention. Complications are nonetheless possible after these procedures, and it would be irresponsible to direct patients to a tool that could not safely identify high‐risk symptoms and point those patients to appropriate follow‐up. In this regard, AI chatbots again show promise, but it is important to note that not all chatbots appear equally safe. ChatGPT was the only queried model that provided adequate responses to all Red Flag Questions (as judged by mean EOQ = 1 across all prompts). Similarly, ChatGPT was the only model to produce highly accurate responses (GQS ≥ 4) for all Red Flag Questions. Again, these findings support the importance of directing postoperative patients toward validated AI models.

Provided responses are accurate and safe, they must also be easily understood by patients to truly be useful in clinical practice. In the United States, the average reading ability is approximately at the eighth‐grade level.^30^ To ensure that most patients are able to understand written educational material, the American Medical Association and National Institutes of Health recommend that patient information be provided at or below a sixth‐grade reading level.^30^ No chatbot in the present study met this mark: even the most‐readable chatbot provided responses above the high‐school level on average.^18^ ChatGPT responses notably scored above the collegiate level on average (median FK > 14). While prior studies have demonstrated that chatbots can significantly reduce the grade level of responses with prompting (eg, “Please present your answers at or below the sixth‐grade United States academic reading level”), this study was designed to capture the most realistic responses patients would receive and therefore did not utilize such prompting.^18^ Readability is clearly an important target for development as chatbot technology advances.

Regardless of the quality of responses, chatbots only have the potential to impact postoperative communication if surgeons are open to their adoption. This study implemented an Expert Opinion Question to assess surgeon comfort with using AI in place of human responders. On average, surgeons approved of chatbot responses in this capacity (overall mean EOQ > 0.5). The superiority of specific chatbots is again seen in this data: ChatGPT produced only 1 unanimously unsatisfactory response and 1 contested response. No other chatbot performed as strongly; Gemini, for instance, produced responses experts were uncomfortable with almost 19% of the time. Taken together, these findings support the notion that chatbots have practical potential in the postoperative context, but that any AI tool should be vetted by surgeons prior to implementation.

An alternative to directing patients to publicly available, high‐performing chatbots is to design purpose‐built AI models and integrate them into existing patient engagement platforms (ie, patient portals). Dwyer et al conducted a pilot study in ambulatory orthopedic patients utilizing a text‐message‐based conversational AI model, nicknamed “Felix.”^27^ Felix, a natural language‐processing model, was trained with postoperative discharge instructions written by the team's surgeons. Felix was well‐received by patients (80% of the 26 participants found the chatbot helpful), provided accurate and safe responses (handled 79% of questions appropriately, with no patient harm in the study), and helped avoid unnecessary healthcare utilization (48% of participants reported successful reassurance by the chatbot).^27^ A notable area for improvement was the chatbot's ability to answer patient questions independently; Felix often relied on facilitating contact with the care team, handling only 31% of questions independently.^27^ This figure still represents reduced workload for clinical teams, but does demonstrate that further advances are necessary to maximize impact. Similar tools are being piloted in other surgical fields.^31^, ^32^

The present study has a number of strengths relative to the existing literature. First, chart review was used to generate a comprehensive set of patient questions. A reliable and safe chatbot must be able to respond to both common postoperative questions and individualized concerns. The majority of published data focuses only on a set of common concerns generated by the authors.^17^, ^18^, ^19^, ^28^, ^29^, ^33^ Additionally, this study compared responses from 4 different AI models to elucidate their respective strengths and weaknesses. Directly comparing a number of different models is increasingly important as AI technology advances and new chatbots enter the market. Finally, the use of the EOQ in this study helps to contextualize the quality of chatbot responses. Prior studies have demonstrated that chatbots can produce accurate and comprehensive responses, but this study additionally explores surgeons' comfort level with using these tools in practice.^17^, ^28^, ^33^

This study is limited by its retrospective design. Providers and staff responding to calls did not document the content of communication in a standardized way, meaning it is possible that some details of conversations were missed. A prospective design could ensure that all nuance of patient communication is captured. Additionally, there is a degree of subjectivity in the metrics experts used to grade AI responses. In the case of the EOQ, this was by design, but with regards to the GQS, a future study could utilize a standardized rubric to more objectively assess quality.

It is important to note that in the present cohort, 27% of patient communication required intervention. Chatbots, at least in their current state of development, are not equipped to provide prescriptions, referrals, or appointments. Therefore, despite promising results, chatbots cannot, and likely should not, replace all postoperative patient communication. Perhaps their greatest potential is as a first‐line triage tool.^27^, ^34^, ^35^ Chatbots’ ability to triage can be expected to improve as technology improves and new features, such as image review, are implemented.^36^

The chatbots examined in this study rely on large language model (LLM) technology, meaning they are trained on large, broad datasets, the content of which clinicians cannot fully audit.^33^ Although ChatGPT overwhelmingly provided accurate information in this study, the fact remains that ChatGPT, like all LLMs, can produce inaccurate responses, particularly if drawing on inaccurate information.^33^, ^37^ To mitigate this patient safety risk, AI models based on retrieval‐augmented generation (RAG), which function similarly to LLMs but draw from curated datasets, are being developed.^37^, ^38^ OpenEvidence (Cambridge, MA) is one such RAG that draws specifically on medical literature.^38^

## Conclusions

Patient calls are common after functional rhinoplasty. Most can be managed with reassurance. Chatbots, especially ChatGPT, provide reliable responses, which may improve satisfaction and reduce workload without compromising safety. Future development should focus on readability.

## Author Contributions

**W. Jack Palmer**, design, conduct, analysis, and presentation of research; **Dana Michlin**, conduct, analysis, and presentation; **Leonard Estephan**, conduct, analysis, and presentation; **Jason Tasoulas**, conduct, analysis, and presentation; **Khashayar Arianpour**, design, conduct, analysis, and presentation; **Harleen K. Sethi**, conduct, analysis, and presentation; **Daniel J. Campbell**, design, analysis, and presentation; **Howard Krein**, design, conduct, analysis, and presentation; **Ryan Heffelfinger**, design, conduct, analysis, and presentation.

## Disclosures

**Competing interests:** None.

### Funding source

None.
